# An Integrated Review of Emoticons in Computer-Mediated Communication

**DOI:** 10.3389/fpsyg.2016.02061

**Published:** 2017-01-06

**Authors:** Nerea Aldunate, Roberto González-Ibáñez

**Affiliations:** ^1^Escuela de Psicología, Universidad de Santiago de ChileSantiago, Chile; ^2^Departamento de Ingeniería Informática, Universidad de Santiago de ChileSantiago, Chile

**Keywords:** emoticons, emotional expressivity, computer-mediated communication, language comprehension, neuroscience

## Abstract

Facial expressions constitute a rich source of non-verbal cues in face-to-face communication. They provide interlocutors with resources to express and interpret verbal messages, which may affect their cognitive and emotional processing. Contrarily, computer-mediated communication (CMC), particularly text-based communication, is limited to the use of symbols to convey a message, where facial expressions cannot be transmitted naturally. In this scenario, people use emoticons as paralinguistic cues to convey emotional meaning. Research has shown that emoticons contribute to a greater social presence as a result of the enrichment of text-based communication channels. Additionally, emoticons constitute a valuable resource for language comprehension by providing expressivity to text messages. The latter findings have been supported by studies in neuroscience showing that particular brain regions involved in emotional processing are also activated when people are exposed to emoticons. To reach an integrated understanding of the influence of emoticons in human communication on both socio-cognitive and neural levels, we review the literature on emoticons in three different areas. First, we present relevant literature on emoticons in CMC. Second, we study the influence of emoticons in language comprehension. Finally, we show the incipient research in neuroscience on this topic. This mini review reveals that, while there are plenty of studies on the influence of emoticons in communication from a social psychology perspective, little is known about the neurocognitive basis of the effects of emoticons on communication dynamics.

## Introduction

Emotions are one of the most important information signals in social cognition (Pessoa, 2009). This information usually comes from facial expressions in face-to-face interactions. However, today people use alternative communication channels – like text-based communication – to interact with others when they are not physically present. In such cases, facial information is not always available. The lack of affective signals and pragmatic information in human communication has been associated with difficulty in understanding other people (Kiesler et al., 1984). In computer-mediated communication (CMC), particularly in text-based communication, face-related information has been partially substituted by emoticons, which enable interlocutors to express different emotional states by using text-based representations of facial expressions. Although emoticons are unnatural, iconic, and static representations of human facial expressions, they have become a popular resource to enrich text-based communication since 1982 (Vincent and Fortunati, 2009). Beyond their uses and impacts, little is known about how emoticons are processed in CMC at the brain level.

In this mini review, we examine socio-cognitive and neuroscience research on the evidence of psychological effects of emoticons in CMC and their processing in human cognition. This article is structured in four parts. The first part presents a review of relevant literature on the importance of facial expressions in communication. Second, the importance of emoticons in text-based communication, social presence, and language processing is discussed. Third, research on neuroscience and emoticons is reviewed. Finally, we conclude with a discussion about future research on this topic and practical implications for cognitive sciences.

## The Importance of Facial Expressions in Communication

Natural interactions in human communication are full of contextual cues, such as gestures, prosody, and facial expressions, which are processed for common understanding. Some theories about human interactions have considered that meaning comprehension requires an ecological context and the use of expressive non-verbal tools to enrich communication, which is the case of face-to-face contexts (Hörmann, 1981).

Facial expressions are considered one of the most important cues in human communication. Evidence suggests that from birth, human beings prefer facial expressions over other types of stimuli (Goren et al., 1975; Johnson et al., 1991). The face is one of the most visible and complex sources of information about the emotional states of individuals. Faces are integrated into comprehension processes during social interactions and communication. In fact, most human beings have a special capacity to process them with specific mechanisms (Ekman et al., 2013). For example, the process of face perception involves a complex neural network that enables people to recognize aspects of facial structure of others and to communicate socially using the perception of facial movements (Haxby et al., 2000, 2002).

In natural conditions, facial expressions and other contextual cues inform individuals about the affective states of the participants in the communication process. However, during the last decades, alternative communication media have proliferated (e.g., desktop computers and smartphones) in developed countries (Poushter et al., 2015). In particular, in text-based CMC there is a physical gap that implies a loss of contextual information (e.g., facial expressions). This aspect constitutes a difficulty for CMC, especially for communication with figurative language (Sarbaugh-Thompson and Feldman, 1998), which may have negative (Sproull and Kiesler, 1986; Okdie et al., 2011) or positive (Walther, 1995, 1996; Bargh et al., 2002) effects on human relationships as a result of the lack of nonverbal and contextual cues.

The lack of face-to-face interactions and therefore the absence of regulating feedback, affect interactions at different levels. It also causes people to behave differently while interacting in some CMC contexts. For example, it is known that when small groups interact face-to-face in a problem-solving discussion, they are more likely to reach agreement than when they communicate in a computerized conference (Hiltz et al., 1986). On the other hand, self-awareness has been reported to be lower in face-to-face communication than in CMC (Matheson and Zanna, 1988, 1990). These differences are due to difficulties in communicative coordination between people without a context that provides informational feedback, cues for controlling discussion, and lack of nonverbal involvement (Kiesler et al., 1984).

## The Importance of Emoticons in Text-Based Communication

Two factors that influence the success of a communication channel are its “social presence” and its “richness.” On the one hand, social presence (Short et al., 1976; Gunawardena and Zittle, 1997) refers to the degree to which a channel can be used to transmit relational information that increases the perception of human interaction with non-verbal cues such as facial expressions (Short et al., 1976). For instance, text-based CMC like chats or emails, have less social presence than face-to-face communication because they lack social non-verbal cues for the interactions. On the other hand, media richness (Daft and Lengel, 1986) refers to the degree of adequacy of a channel to the communication context and goals in order to reduce uncertainty and equivocality. For example, managers may prefer face-to-face group meetings over email exchange in order to avoid misinterpretations of messages when working on a task. In this scenario, face-to-face communication, which is equipped with a variety of resources, such as facial expressions and prosody, is considered to be richer than text-based communication.

Despite its levels of social presence and richness, text-based communication often has a social function; however, it needs more expressive tools to enrich and socialize interactions. One way text-based CMC users have made their conversations more dynamic, conveying more social presence and richness has been through the use of emoticons. Emoticons can be considered a humanized tool for emotional expressiveness that replaces cues that are naturally present in face-to-face and voice-to-voice communication. Emoticons provide enjoyment and more information to the interaction (Huang et al., 2008) and they also enhance the comprehension of messages (Walther and D’Addario, 2001) with a more accurate perception of emotions, attitudes, and intentions during text-based communication (Lo, 2008).

While there is quite abundant evidence on how emoticons are used in text-based CMC, little is known about how they are processed at the cognitive and brain level. This knowledge could help to explain the effects of emoticons on text-based communication. In this sense, both cognitive science and neuroscience could contribute to address this question. On the one hand, research in cognitive science related to language processing constitutes a valuable framework for understanding how emoticons can influence the interpretation of messages. On the other hand, research in neuroscience provides further evidence on how the brain operates in CMC contexts, especially when interlocutors perceive affective information on schematic faces integrated with language messages.

## Influence of Emoticons on Language Comprehension

Facial expressions are naturally present in face-to-face communication, however, in mediated communication, particularly in CMC, communication of facial expressions may be limited to the richness of the underlying communication channel (Daft and Lengel, 1986). Emoticons can be defined as facial expression surrogates, which correspond to symbolic, lexical, or graphical resources used in written communication to convey emotional expressions. In this sense, emoticons can be used as paralinguistic cues of an utterance, which in written communication can help writers and readers to elucidate meaning (Schuller and Batliner, 2013).

Emoticons have been described, in some cases, as useful resources in text-based mediated communication (Rivera et al., 1996). They have provided people with an alternative way to express their emotions, feelings, and mood changes to others in restricted communication channels such as text-based ones. Emoticons have been important in increasing social presence in text-based interactions, where communication tends to be cold without affective cues. For example, a study has shown that in socio-emotional contexts, emoticons are more commonly used than in task-oriented ones (Derks et al., 2007b). Likewise, the same authors also showed that emoticons are more common in interactions between friends than between strangers, and in positive contexts than in negative ones (Derks et al., 2008).

It has been stated that emoticons can play a central role in interpreting text messages in CMC (Walther and D’Addario, 2001). In this context, Derks et al. (2007a) and Lo (2008) found that text messages can be strengthened by using emoticons. Emoticons can also change the direction of a message to the opposite direction, which is the case of sarcastic utterances. A related study by Filik et al. (2015) showed that emoticons are more expressive than punctuation marks in the disambiguation of sarcastic utterances. Along the same lines, González-Ibáñez et al. (2011) and Muresan et al. (2016) showed that emoticons in short messages like those found on microblogging platforms (e.g., Twitter) are particularly helpful for humans and machines in classifying sarcastic, non-sarcastic, positive, and negative messages. For example, consider the following written sentence: “I love the customer service of this company.” Without a context and lack of verbal cues, it is likely that its interpretation is literal. However, with the incorporation of traditional emoticons (i.e. :) and :( ) at the end of the sentence, its intentional meaning (literal and sarcastic, respectively) is clarified.

Contrary to the above studies, results from an experiment conducted by Walther and D’Addario (2001) showed that the contribution of emoticons in interpreting messages is limited. However, the authors found consistency in the interpretation of negatively biased messages as a result of both negative emoticons and negative verbal content.

In addition to message interpretation, Thompson et al. (2016) found that emoticons have the potential to reduce negative responses that are typically experienced when exposed to ironic texts. It is important to note that these findings are contextualized in an artificial setting in which electrodermal activity (for arousal) and electromyography (for facial expressions) were used to measure participants’ reactions. Overall, the authors found that the presence of emoticons increased arousal levels, reduced frowning expressions, and enhanced smiling.

The above articles illustrate the active role that emoticons can play in language comprehension, specifically in text message interpretation. Although simple in expressions, emoticons have the potential to enrich verbal expressions acting as substitutes of richer forms, such as facial expressions and prosody, which are naturally present in face-to-face interactions.

## What Does Neuroscience Tell Us About Emoticons?

Recently, neuroscientists have been studying neural processing during emoticon perception. Different techniques have been chosen to study emoticon processing. One of these has been functional Magnetic Resonance Imaging (fMRI), which provides information on the brain areas involved in particular processing with a high spatial resolution. The other technique is electroencephalography (EEG), which provides high temporal resolution. In EEG studies, there is a specific technique known as event related potentials (ERPs), which provides information about the time course of the processing and its characteristics after a specific stimulation.

### fMRI Studies

One question that has driven research is whether emoticons activate the same brain areas that faces do. One of the structures involved in face processing is the right fusiform gyrus, which participates in face recognition (Kanwisher and Yovelm, 2006) and presents dysfunctional activation in patients with prosopagnosia, which refers to the incapacity to recognize faces (Barton et al., 2002).

Initial fMRI studies indicated that isolated emoticons do not activate the same brain regions in the processing of human faces. However, they activate the same areas involved in emotional discrimination, like the right inferior frontal gyrus. Yuasa et al. (2006) showed that in an emotional valence decision task, human faces activate the right fusiform gyrus, right inferior frontal gyrus, right middle frontal gyrus, and right inferior parietal lobe. Conversely, Japanese emoticons did not activate the right fusiform gyrus, but they activated the right inferior frontal gyrus. These findings suggest that these kinds of emoticons may be associated with emotional discrimination. In a posterior study, Yuasa et al. (2011) investigated brain activations during the exposure to emoticons presented after sentences. Results showed that emoticons are associated with nonverbal information processing and that the brain areas involved in verbal and nonverbal information processing are more active when sentences are followed by emoticons than when they are not. Specifically, they found that, although the right fusiform gyrus was not activated when sentences with emoticons were presented, the right inferior frontal gyrus was activated. This finding indicates that emotional discrimination based on emoticon perception is similar to other non-verbal cues like facial expressions or speech prosody.

In another study, Shin et al. (2008) compared brain activity for pictures of real faces and real houses with their corresponding schematic icons to observe the neural basis for iconic symbol recognition. For this purpose, they used emoticons as face icons. The results of this study showed more cortical brain activation than subcortical for icons, involving the frontal and parietal cortex, and the temporo-occipital junction in the ventral pathway – implied in the know-what processes, which are related to object recognition. The authors suggest that this activation corresponds to neural correlates of visual concept formation, distinguishing it from the perception of concrete visual objects. Specifically, they observed a bilateral activation of the fusiform gyrus during the exposure to both emoticons and house icons. Such activation could be associated with the characteristics of the icons, which share aspects of words and objects. The authors also observed activation in a region known as Visual Word Form Area, which is a specific area of he left fusiform gyrus that responds to word’s letters (Cohen et al., 2002) and participates in “integrating the shape elements into a meaningful whole” (Starrfelt and Gerlach, 2007; Shin et al., 2008, p. 303). Unlike emoticons, faces activated the inferior occipital cortex, which participates in face information processing at the individual level (Gauthier et al., 2000), and this activity decreased along the ventral pathway.

Recently, the activity in the fusiform gyrus for emotional words and emoticons was observed in patients with autism spectrum disorder (ASD) (Han et al., 2014). These patients have a social dysfunction due to different brain pattern alterations. For example, patients with ASD do not present activation in the fusiform gyrus when they see emotional facial expressions (Critchley et al., 2000; Pierce et al., 2001). Moreover, patients with ASD do not show activation in the right fusiform gyrus in the discrimination of facial expressions (Schultz et al., 2000). In the study of Han et al. (2014), patients with ASD and healthy participants were exposed to emotional words and emoticons to observe the activation of the fusiform gyrus. They observed an increment in the activity of the fusiform gyrus for emotional words, which led the authors to hypothesize that this result could be a manifestation of a compensatory mechanism to control social information processing. Nevertheless, further analyses of brain activation during the presentation of emoticons revealed a lower activation in the fusiform gyrus. This result suggests that word descriptions are more familiar with emotional processing than facial expressions in ASD patients.

### EEG Studies

Face processing has been studied with EEG showing the N170 ERP for face perception. This component is a negative-going deflection in the occipito-temporal cortex with a maximum peak around 170 ms after the presentation of a face and it is related to the configurational processing of faces (Bentin et al., 1996; Allison et al., 1999; McCarthy et al., 1999). It is known that this component appears even if faces are schematic (Sagiv and Bentin, 2001; Babiloni et al., 2010).

Today, there are very few studies about emoticon processing with EEG recording. Churches et al. (2014) obtained similar ERPs (N170) for typographic smiley emoticons “:-)” and faces, suggesting similarities in emoticon and face perception. A study by Comesaña et al. (2013) suggests that processing of emoticons is more privileged than that of the word to which they refer to. In particular, the authors showed a priming effect with masked emoticons in word processing with modulations in N2 and late positive component (LPC), which is not present by masked affective words. It is known that positive words with high positive arousal level influence LPC (Gibbons, 2009), reflecting the activation of motivational and attentional brain systems by emotional stimuli and the initial memory storage during the processing of affective information (Cuthbert et al., 2000). It is important to note that this component is related to the stimuli’s arousal and not to its valence (Hinojosa et al., 2009).

## Concluding Thoughts

At present, humans relations are rapidly evolving through the use of electronic means. This fact has involved an increasing use of alternative cues to communicate emotional states in computer-mediated contexts. Emoticon, as one of these alternative cues, can change people’s dispositions in the comprehension processes or interactions with others. Moreover, they can affect decisions, mood, or perspective of the conversation.

The use of emoticons is important in CMC because they enable greater emotional expressiveness in absence of context. Not only do emoticons provide more fun to the interaction (e.g., Huang et al., 2008; Thompson et al., 2016), but they also facilitate disambiguation of messages, enabling better comprehension (Lo, 2008), therefore they are an expressive resource. Although this is important, there is little evidence on the cognitive and brain dimensions on how these emoticons are processed in human communication. Today, studies have focused on processing structural characteristics of emoticons and their relationship with the processing of emotional information and human faces. However, further research toward understanding the processing of emoticons within the dynamics of text-based communications is required. This would enable in-depth understanding of how affectivity unfolds in CMC interactions and how this affective dimension affects language understanding.

Additionally, it is important to note that not all emoticons have the same degree of humanization. For example, emojis are emoticons that are more human than typographic in nature. Research on the effects of different types of emoticons according to their structural characteristics (e.g., emojis, typographic, Japanese, etc.) may be useful for developing communication technology to favor social presence and the affectivity that characterizes communication in social interactions.

## Author Contributions

All authors listed, have made substantial, direct and intellectual contribution to the work, and approved it for publication.

## Conflict of Interest Statement

The authors declare that the research was conducted in the absence of any commercial or financial relationships that could be construed as a potential conflict of interest.
